# The Evolution of the Steps Program, 2003-2010: Transforming the Federal Public Health Practice of Chronic Disease Prevention

**DOI:** 10.5888/pcd9.110220

**Published:** 2012-02-02

**Authors:** Phyllis Nichols, Ann Ussery-Hall, Shannon Griffin-Blake, Alyssa Easton

**Affiliations:** Centers for Disease Control and Prevention; The Ginn Group, Atlanta, Georgia; Centers for Disease Control and Prevention, Atlanta, Georgia; Centers for Disease Control and Prevention, Atlanta, Georgia

## Abstract

The Steps program, formerly known as Steps to a HealthierUS, was the first Centers for Disease Control and Prevention (CDC) program to support a community-based, integrated approach to chronic disease prevention. Steps interventions addressed both diseases and risk factors, focusing on the 3 leading causes of preventable deaths in the United States — tobacco use, poor nutrition, and physical inactivity — and the associated chronic conditions of asthma, diabetes, and obesity. When Steps shifted from interventions focused on individual health-risk behaviors to the implementation of policy, systems, and environmental changes, the program became an integral part of changing the way CDC addressed chronic disease prevention. In this article, we describe the shift in intervention strategies that occurred among Steps communities, the model that was developed as Steps evolved, common interventions implemented before and after the shift in approach, challenges experienced by Steps communities, and CDC programs that were modeled after Steps.

## Introduction

To address the 3 leading causes of preventable deaths in the United States — tobacco use, poor nutrition, and physical inactivity and the associated chronic conditions of asthma, diabetes, and obesity, the — Centers for Disease Control and Prevention (CDC) implemented the Steps program, formerly known as Steps to a HealthierUS, in 2003. At that time, tobacco use, the most avoidable actual cause of death, caused illness among 8.6 million people in the United States (1). Poor diet and lack of exercise, the second and third actual causes of preventable death, were leading contributors to an obesity epidemic (2). Asthma and type 2 diabetes rates had also reached epidemic proportions (3,4). Prevalence of disabling asthma among children (categorized by the National Health Interview Survey as asthma that limits children's ability to perform usual activities) had increased 232% during the past 4 decades (5) and diabetes rates had more than doubled (4). Researchers predicted that one-third of all people born in 2000 would develop type 2 diabetes during their lifetime (4). Obesity rates also doubled from 1980 through 2002 (6), and by 2002, more than 22% of adults were obese (defined as body mass index ≥30 kg/m^2^) (7). Comprehensive efforts to combat unhealthy behaviors that contributed to the rising rates of chronic disease were needed.

## Background

**Figure 1.**
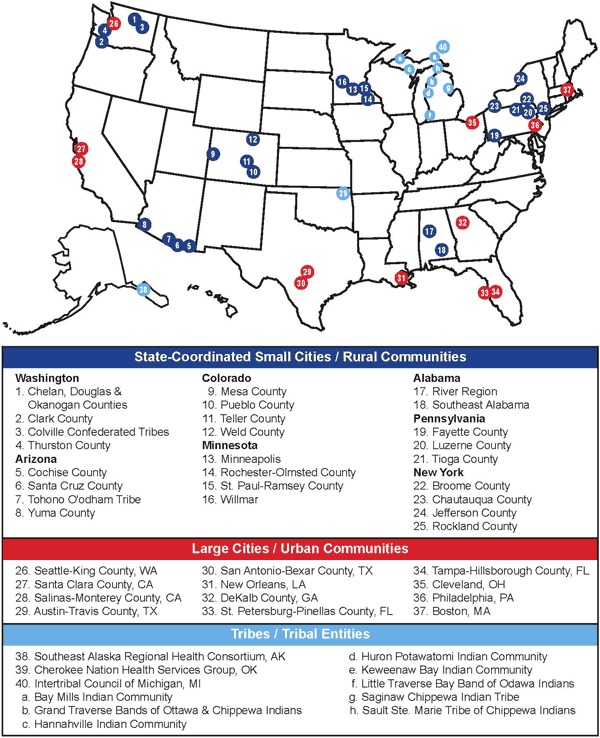
The Steps program map of communities by categories of eligibility.

Steps comprised 2 cooperative agreements coordinated by CDC and funded by the Department of Health and Human Services (HHS) in fiscal years 2003 and 2004. Forty communities (Figure 1) and 1 national partner (YMCA of the USA) were selected for funding according to the following criteria: ability to demonstrate public health capacity and expertise to implement Steps initiatives; demonstrated need based on high rates of asthma, diabetes, and obesity compared with the national average; existence of disparate populations with inadequate access to quality care; and proposed program goals and objectives. In fiscal year 2003, HHS distributed $13.6 million to12 grantees representing 24 communities. In fiscal year 2004, HHS distributed an additional $35.8 million to increase support to existing communities and fund 10 additional grantees representing 16 communities. In fiscal year 2004, HHS also distributed $2 million to the YMCA of the USA to partner with Steps communities and increase the reach of Steps-funded efforts.

Whereas most programs at CDC addressed a specific disease, condition, or risk factor in a particular setting and among a certain population until program funding ended, Steps interventions encompassed multiple diseases and risk factors, served entire communities, and were designed to be sustainable beyond federal funding.

Steps interventions addressed all levels of the social ecological model (SEM) (8) — individual, interpersonal, organizational, community, and public policy — to prevent and control health outcomes related to asthma, diabetes, and obesity.

## Shift in Intervention Strategies

In Steps' earlier years (2003-2006), communities primarily implemented evidence-based interventions focused on changing health behavior at the individual level. Interventions were aimed at increasing awareness and educating participants about chronic disease prevention, such as diabetes self-management training, nutrition education, and smoking cessation classes. Steps communities came to the common realization that, because characteristics of a healthy community were interdependent, a comprehensive approach was required to maximize Steps' potential. These characteristics included the built environment (eg, sidewalks, bicycle paths, and walking trails that promote physical activity); accessibility and availability of population-based health programs (eg, interventions offered in community clinics); access to healthy foods in restaurants, cafeterias, convenience stores, and farmers' markets; and health behavior (eg, walking or bicycling to destinations, eating healthy foods). It became evident that a systems approach involving policy and environmental change strategies was needed, because interventions aimed at individuals could reach only a small percentage of the population at a time. Steps communities reported that these programs reached 100 people or fewer per intervention, whereas a typical systems or policy approach could reach hundreds to hundreds of thousands of people in the same period.

**Figure 2.**
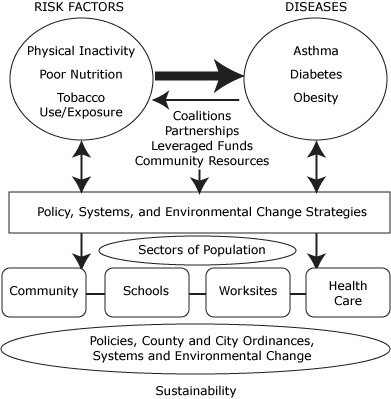
The Steps Model.

Steps communities mobilized key decision makers and community members to increase awareness about the need for altering the social and built environment to support healthy living. They strengthened their coalitions to include key business leaders, county and city planners, transportation professionals, and local elected officials to help foster changes to the local food and physical environments. Grassroots movements to pass local ordinances such as trans-fat bans (9-11), menu-labeling legislation (12,13), and smoke-free housing (14) emerged, and Steps communities became leaders in community-level change. By the end of 2006, Steps communities embraced a model for chronic disease prevention that focused on the implementation of sustainable, practice- or evidence-based policy, systems, and environmental (PSE) changes to prevent and control rising rates of asthma, diabetes, and obesity. By 2008, Steps' approach was recognized in a report by Trust for America's Health as a successful community-based public health intervention approach that could be cost-effective and estimated that the program could save $16 billion in US health care costs annually within 5 years (15). CDC developed the Steps Model (Figure 2) to highlight Steps' PSE change approach.

## Measurement

Performance measurement was the centerpiece of evaluating Steps communities as a whole. Together, Steps program staff and stakeholders developed a set of 18 performance measures (8 implementation measures and 10 outcome measures) and 44 indicators (for implementation and outcome measures) (Appendix).

Steps communities reported their progress annually during 2003 through 2010, using the performance measures reporting system. Process measures summarized the communities' progress in using evidence-based strategies, leveraging partner resources to support Steps initiatives, and integrating program components.

The communities tracked implementation measures and related indicators using community-specific data collection methods and tools. Implementation indicators included numbers of interventions that addressed multiple risk factors in different sectors and at multiple levels of the SEM.

Outcome measures and related indicators included physical activity and nutrition behaviors, access to and quality of clinical services, and management of chronic diseases and conditions (Appendix). Outcome indicators were tracked through existing surveillance mechanisms, including the Youth Risk Behavior Surveillance System and Behavioral Risk Factor Surveillance System. CDC combined and analyzed performance-measure data annually and used aggregate data summaries to assess overall progress of the Steps program (16-18).

## Interventions Summary

The following results were obtained from performance measure reports CDC received from the 40 Steps communities. For interventions implemented during 2003 through 2006, communities reported generic names for interventions, evidence-based sources, population sector receiving the intervention, and diseases or risk factors each intervention addressed. For example, a community may have reported that it implemented diabetes self-management education classes (intervention) on the basis of evidence of effectiveness reported in the *Community Guide to Preventive Services* (source of evidence) for community members (sector) diagnosed with diabetes (disease) who were overweight or obese (risk factor). By 2006, Steps communities had implemented approximately 1,500 interventions; 94% of the interventions were evidence-based, and 74% addressed 2 or more diseases or risk factors. The most common interventions implemented during 2003 through 2006 targeted individuals (Table 1).

Beginning in 2007, when Steps communities shifted their focus to the implementation of PSE change strategies, performance measure reporting also included the number and type of strategies implemented in each sector. By this time, all of the communities had implemented interventions that addressed 2 or more diseases or risk factors; 85% addressed all risk factors, 93% implemented at least 1 intervention at all levels of the SEM, and 85% addressed access to health care or quality of care. Steps communities implemented approximately 2,300 PSE change strategies during 2007 through 2010 (Table 2).

### PSE change strategy examples

**Fruit and vegetable promotion**

A large urban area implemented a countywide community gardens project to increase access to affordable local produce. This Steps community partnered with a university cooperative extension service to provide outreach and training to residents interested in starting a community garden. The project resulted in the creation of 31 neighborhood produce gardens with 1,085 participating gardeners. Partnerships were established with food pantries and other emergency food providers, and thousands of pounds of produce were donated. The project also led to policy changes; project staff worked with the city council and city planning agency to pass new zoning legislation. Their success led to implementation of 7 additional gardens that received a total of $30,000 in grants from local foundations and businesses.

**School-based health education policy**

One of the school-based interventions implemented by a Steps community involved a policy that added the health education curriculum Planet Health to the academic curriculum in 16 middle schools, reaching 4,200 students. The Planet Health curriculum was chosen because it has been shown to decrease rates of obesity and disordered eating behaviors among girls, significantly reduce time spent watching television for boys and girls, and increase fruit and vegetable intake, knowledge of nutrition, and healthy activities among students (19). Students learned about the benefits of good nutrition and physical activity while building skills in language arts, health, math, science, and social studies. School teachers and staff received annual training on integrating this curriculum into daily classroom learning activities. After 1 year of using this curriculum, approximately 90% of teachers reported that it had a positive effect on both students' and their own health habits.

**Comprehensive worksite wellness initiative**

One Steps community established a "Healthiest Business Challenge" that awarded points for worksite participation in initiatives such as walking programs, healthy-meeting food policies, stairwell-use campaigns, and establishment or modification of written smoke-free worksite policies. Challenge winners received awards during a Chamber of Commerce meeting and recognition in a local business journal. During the first 3 years of the initiative, 34 businesses and approximately 6,750 employees completed the challenge. Approximately 80% of the participating businesses implemented or upgraded at least 1 worksite policy related to nutrition, physical activity, or smoking cessation. This initiative is now being conducted throughout the state (20).

**Policy for health care providers**

A partnership between a Steps community and a hospital resulted in implementation of a policy requiring health care providers to focus on patients' tobacco use and cessation efforts. Providers from various disciplines were trained to follow the 5 A's model (21), a 2-minute strategy shown to increase tobacco-use cessation by encouraging health care providers to 1) ask patients abouat tobacco use, 2) advise them to quit, 3) assess patients' willingness to quit, 4) assist in cessation attempts, and 5) arrange for patient follow-up. Providers were also encouraged to refer patients to the state quitline, where intensive counseling was provided. Approximately 550 health care providers were trained on the 2-minute intervention, and the hospital changed its patient intake and education forms to reflect the new process. As a result of the new policy, total calls to the quitline from health care provider referrals quadrupled in 1 year, and calls increased 50% compared with those made in a neighboring county with similar demographics.

### PSE challenges

Researchers have increasingly recognized the importance of PSE change to achieve widespread and sustainable improvements in health behaviors (22-24). They also acknowledge the challenges of implementing strategies (25-28). Successful PSE change strategy implementation often depends on the assumption that voters and stakeholders, who are often large corporate entities, will accept serious changes to the food, school, and built environments. Schools and local governments encounter budgetary barriers to implementing these strategies (eg, mandating physical and nutrition education in schools often requires hiring new staff, offering healthier cafeteria foods costs more, and constructing bicycle paths and sidewalks requires transportation funds that are already limited). Legal and constitutional hurdles can restrict efforts to place limits on advertising of unhealthy products, and contractual concerns may thwart attempts to change vending practices, especially in schools that benefit from proceeds. Steps communities encountered these challenges, yet they were able to collectively implement approximately 2,300 PSE change strategy interventions during 2007 through 2010.

Steps communities also experienced challenges identifying short-term health outcomes. Long-term health outcomes associated with their work are unknown. Systemwide changes in health behavior (eg, students at an elementary school who collectively increased their fruit and vegetable consumption) were not always observed in the short term. Data and descriptions of interventions were based on self-report, and although validity and reliability concerns associated with this method of data collection arose, CDC staff conducted comprehensive, in-person site visits and validated intervention descriptions and the existence of all reported interventions.

## Conclusion

Steps was instrumental in transforming the way CDC addresses chronic disease prevention. It was the first CDC program to fund local jurisdictions to implement PSE change strategies. Communities throughout the United States have replicated Steps policy initiatives, such as menu-labeling requirements, trans-fat bans, and smoke-free housing. In Philadelphia, the Steps Program supported the most comprehensive menu-labeling legislation that existed in 2009 (29). In 2008, Boston Steps helped the city became one of the first in the nation to ban the use of artificial trans-fats in restaurants and grocery stores (10). The Steps program in Clark County, Washington, helped implement one of the first countywide smoke-free housing policies for government-owned residences in the United States (14).

Since 2008, CDC has been designing and implementing programs that support PSE change strategy based on the Steps Model, including Strategic Alliance for Health in 2008 and Communities Putting Prevention to Work in 2010. Most recently, CDC launched the Community Transformation Grant program to increase opportunities for state-supported communities, counties, cities, territories, and tribes to implement integrated, sustainable PSE change-focused strategies, based in part on the Steps Model.

Although policies, programs, and other interventions that promote healthy eating, physical activity, and smoke-free public places have resulted from the growing recognition of their importance, they are often being implemented despite the lack of evidence for their effectiveness. As CDC continues to fund communities to do this work, the evidence base for these types of strategies will grow. Although CDC has supported publications that document some Steps communities' successes and lessons learned (30-34), more reports in the scientific literature are needed.

## Acknowledgments

We acknowledge the expert editorial contributions of Kristen Folsom.

## Figures and Tables

**Table 1:** The Most Common Interventions Implemented by Steps Communities, 2003-2006

**Sector/Intervention**	No. of Interventions
**Community**	
Distribution of health education materials	116
Diabetes education classes	84
Exercise classes	72
Nutrition education classes	60
Asthma education classes	44
Faith-based wellness trainings	40
Health fairs	40
Smoking cessation classes	32
Healthy cooking classes	28
Stop smoking call centers/quitlines	20
Diabetes support groups	20
**School**	
Asthma management (for students with asthma, school nurses/staff)	71
Fitness programs to measure individual student fitness	64
Nutrition education materials	60
Nutrition guidelines for cafeteria staff	56
Health fairs	36
Coordinated School Health	32
Students Working Against Tobacco (SWAT) teams	28
Tobacco cessation classes	20
School produce gardens	16
Diabetes management (for students with diabetes, school nurses/staff)	12
Walk to School Day	12
**Worksite**	
Worksite wellness programs	76
Healthy meetings	44
Weight management classes	40
Health fairs	40
Stairwell promotion	36
Tobacco-free worksite initiative	28
Smoking cessation programs	24
Pedometer distribution	24
Reduced-price or free gym memberships	16
Space for exercise on site	12
Lactation rooms on site	8
**Health care **	
Chronic disease management (asthma, diabetes)	52
Provider education	48
Tobacco-use cessation classes	28
One-on-one dietary counseling for people with diabetes	24
Expanded use of community health workers	24
Provider reminder kits	24
Improved access to health care	20
**Total**	1,531

**Table 2:** Most Common Policy, Systems, and Environmental (PSE) Change Strategies Implemented by Steps Communities, 2007-2010

**Sector/PSE Change Strategy**	No. per Strategy
**Community**	
Fruit and vegetable promotion (nonspecific)	240
Access to community health facilities	87
New trails or walking paths	63
Walkability/bikeability assessments	61
Grocery food/restaurant menu labeling	60
Trail promotions	60
Smoke-free parks (policies and ordinances)	57
Farmers' markets	51
Community gardens	48
Parks/playground access	42
Food sustainability	38
Smoke-free housing	36
Safe Routes to School	36
Zoning projects/plans	29
Traffic calming measures	18
Healthy vending (not schools or worksites)	18
**School**	
Nutrition education curriculum	196
Healthy cafeteria/vending food options	141
Physical education 3-5 days/week	114
Healthy food/beverage options at school events	76
Asthma management policies	72
Increased recess time (with physical activity options)	46
Tobacco-free campuses	31
School gardens	29
Diabetes management	25
Tobacco cessation programs	24
**Worksite**	
Health risk assessment	49
Stairwell promotion	47
Healthy vending machine policy	45
Healthy meeting food policy	41
Tobacco-free worksite	33
Space for exercise on-site	32
Smoking cessation program	30
Reduced-price or free gym membership	23
Paid/flex work time for exercise	23
Insurance break for risk reduction	7
Breastfeeding policy	5
**Health care **	
Counseling on risk factors (physical activity, nutrition, smoking)	112
Chronic care model	72
Tobacco cessation	41
Community health workers	34
Reimbursement of preventive care	10
**Total**	2,302

## Appendix: Steps Program Performance Measures and Indicators

**Table:**

**Program Implementation Measures (I)**	
I-1 Align budget with program goals and intended outcomes.	I-1.1 Fiscal resources allocated to address Steps focus areas and key health outcomes
I-2 Ensure community objectives and activities are supportive of state plans but do not duplicate interventions or activities.	I-2.1 Objectives and activities linked to the work of state programs to prevent and controlobesity, diabetes, asthma, or associated risk factors
I-3 Expand available resources by engaging in public-private ventures and securing foundation grants, other public funding, and in-kind contributions.	I-3.1 Resources secured to supplement funds received via the Steps Program (eg, nonfederal grants and in-kind support)
I-4 Participate in coordinated monitoring and evaluation activities, including data collection and reporting on common performance measures and planning and implementing national evaluation activities.	I-4.1 Submission of data on core performance measures according to established schedule I-4.2 Participation in national-level evaluation tasks (eg, sending feedback to Steps Program Office on draft documents, task-specific workgroups, conference calls)
I-5 Expand existing surveillance mechanisms to collect representative Behavioral Risk Factor Surveillance System (BRFSS) and Youth Risk Behavior Surveillance System (YRBSS) data.	I-5.1 Appropriate and representative data collected via BRFSS I-5.2 Appropriate and representative data collected via YRBSS
I-6 Use multiple, evidence-based public health strategies.	I-6.1 Documented evidence for activities related to all the diseases and risk factors of interest to the Steps Program
I-7 Improve integration of program components.	I-7.1 Implementation of 1) interventions that address at least 2 diseases or risk factors and 2) at least 1 intervention at each key sector I-7.2 Implementation of evidence-based interventions that address access to health care, quality of health care, and use of health care I-7.3 Implementation of evidence-based interventions across the socioecological model (ie, individual, interpersonal, organizational, community, and public policy) I-7.4 Partnership with the YMCA of the USA, or its local affiliate, to improve access to places for physical activity I-7.5 Composition and function of Steps Leadership Team (eg, inclusion of nontraditional agencies or partners, state or local categorical programs, key community-based organizations, or representatives of the health care sector) I-7.6 Composition and function of Steps State-Community Management Team (eg, inclusion of nontraditional agencies or partners, state or local categorical programs, key community-based organizations, or representatives of the health care sector) I-7.7 Provision of technical assistance to state-coordinated Steps communities (state only; eg, trainings and evaluation assistance)
I-8 Document that intended populations participate in Steps communities' activities and interventions.	I-8.1 Reach (eg, a tobacco intervention was implemented in an intervention area to serve specific populations identified in the community action plan)
**Outcome Measures (O)**	
O-1 Increased knowledge and awareness about healthy behaviors such as healthful eating, physical activity, and avoiding tobacco use.	O-1.1 Community-specific indicators (eg, knowledge of recommended fruit and vegetable consumption among youth)
O-2 Increased knowledge about getting appropriate preventive screenings.	O-2.1 Community-specific indicators (eg, knowledge of recommended screenings for people with diabetes)
O-3 Increased physical activity and healthful eating for children and adults.	O-3.1 Fruit and vegetable consumption among adults aged 18 or older O-3.2 Fruit and vegetable consumption among youth O-3.3 Recommended physical activity among adults aged 18 or older O-3.4 Recommended physical activity among youth O-3.5 Television viewing among youth
O-4 Increased access to and quality of clinical services for diabetes, asthma, and tobacco use cessation.	O-4.1 Health care access O-4.2 Foot examination for adults aged 18 or older with diabetes O-4.3 Dilated eye examination for adults aged 18 or older with diabetes O-4.4 Glycosylated hemoglobin measurement at least twice a year for adults aged 18 or older with diabetes
O-5 Increased identification of people with prediabetes and diabetes.	O-5.1 Reduce the overall rate of diabetes that is clinically diagnosed among adults O-5.2 Reduce the overall rate of diabetes that is clinically diagnosed among youth
O-6 Improved self-management of diabetes and asthma.	O-6.1 Self blood-glucose monitoring among adults aged 18 or older with diabetes O-6.2 Self foot exam for adults aged 18 or older with diabetesO-6.3 Symptom-free days among adults aged 18 or older with asthma
O-7 Measurable improvements in healthful eating, physical activity, and tobacco use. Indicators include O-3.1–O-3.5, in addition to those at right.	O-7.1 Tobacco-use cessation attempts by adult smokers O-7.2 Tobacco-use cessation attempts by adolescent smokers O-7.3 Cigarette smoking among adults aged 18 or older O-7.4 Cigarette smoking among youth
O-8 Slowed upward trend of overweight and obesity in Steps communities.	O-8.1 Prevalence of overweight or obesity among adults aged 18 or older O-8.2 Obesity prevalence among adults aged 18 or older O-8.3 Overweight prevalence among youth
O-9 Reduced hospitalizations due to diabetes complications and asthma exacerbations.	O-9.1 Hospitalization with asthma among adults aged 18 or older O-9.2 Hospitalization with asthma among youth O-9.3 Hospitalization with diabetes among adults aged 18 or older
O-10 Improved health-related quality of life.	O-10.1 Mean number of healthy days among adults aged 18 or older
